# Factors influencing the implementation of decision support systems for antibiotic prescription in hospitals: a systematic review

**DOI:** 10.1186/s12911-023-02124-4

**Published:** 2023-02-06

**Authors:** Pinar Tokgöz, Jessica Hafner, Christoph Dockweiler

**Affiliations:** School of Life Sciences, Department Digital Health Sciences and Biomedicine, Professorship of Digital Public Health, University of Siegen, 57068 Siegen, Germany

**Keywords:** Resistance, Decision support systems, Antibiotics, Hospitals

## Abstract

**Background:**

Antibiotic resistance is a major health threat. Inappropriate antibiotic use has been shown to be an important determinant of the emergence of antibiotic resistance. Decision support systems for antimicrobial management can support clinicians to optimize antibiotic prescription.

**Objective:**

The aim of this systematic review is to identify factors influencing the implementation of decision support systems for antibiotic prescription in hospitals.

**Methods:**

A systematic search of factors impeding or facilitating successful implementation of decision support systems for antibiotic prescription was performed in January 2022 in the databases PubMed, Web of Science and The Cochrane Library. Only studies were included which comprised decision support systems in hospitals for prescribing antibiotic therapy, published in English with a qualitative, quantitative or mixed-methods study design and between 2011 and 2021. Factors influencing the implementation were identified through text analysis by two reviewers.

**Results:**

A total of 14 publications were identified matching the inclusion criteria. The majority of factors relate to technological and organizational aspects of decision support system implementation. Some factors include the integration of the decision support systems into existing systems, system design, consideration of potential end-users as well as training and support for end-users. In addition, user-related factors, like user attitude towards the system, computer literacy and prior experience with the system seem to be important for successful implementation of decision support systems for antibiotic prescription in hospitals.

**Conclusion:**

The results indicate a broad spectrum of factors of decision support system implementation for antibiotic prescription and contributes to the literature by identifying important organizational as well as user-related factors. Wider organizational dimensions as well as the interaction between user and technology appear important for supporting implementation.

**Supplementary Information:**

The online version contains supplementary material available at 10.1186/s12911-023-02124-4.

## Background

The rise of antibiotic resistance is widely acknowledged to be an increasing threat to global public health. Worldwide 700.000 people die because of antibiotic-resistant infections [1]. The development of antibiotic resistance is mainly promoted by inadequate antibiotic prescribing practices by physicians, such as antibiotic intake in viral infections, incorrect dosing and incorrect dosing cycles [2, 3]. This results in treatment problems such as prolonged hospital stays or a reduced quality of life, which can be challenging for the affected persons as well as for the whole society [4].

Providing antibiotic therapy indicates constant decision making, which requires responsibility and conscientiousness, because it can have a significant impact on patients´ health [5]. Due to the complexity of medical information, decisions are often made under uncertainty as well as with limited previous knowledge and under great time pressure [6]. In addition, medical decisions don’t base solely on medical factors, but also on the manifold organizational processes of the hospital as an institution with a high degree of division of labor [7, 8].

Information technology has become increasingly prevalent in all industries, especially in healthcare. The development of a wide variety of information systems employed to aid clinicians in decision-making [9]. One such system is called decision support systems (DSSs). These are defined as software systems that are used to facilitate decision-making for clinicians by preparing data and making it available to users in a structured and selective manner, in order to support the clinical decision [10]. Classically, DSSs use knowledge systems that rely on if–then rules. Increasingly, machine-learning techniques are used, where large data sets are used to learn from further events and so recognize certain patterns. Both methods are based on artificial intelligence that are often combined in applications [11].

Many studies demonstrate that DSSs can potentially offer considerable support for many aspects of the appropriate use of antibiotics [12] and advantages for reducing toxic drug levels and medication errors as well as costs [13, 14]. While evidence on the technological characteristics of DSSs or the effectiveness on clinical outcomes are widespread [15–17], there is a need of evidence that provides insights into wider social and organizational aspects that needed to accompany successful implementation [18, 19]. Although there are diverse approaches explaining the adoption of DSSs, relevant studies often describe the effects of a DSS on level of disease management without addressing the importance of matching user-related characteristics, the technology being implemented and the organizational circumstances all together [20, 21]. The aim of this systematic review is to identify facilitating and impeding factors of DSS implementation for antibiotic prescription in hospitals at the levels of technology, organization as well as user and by use of a sociotechnical framework.

## Methods

The search, systematization and analysis of literature follows the recommendations of the PRISMA statement [22].

### Search strategy

An extensive search of literature was performed in the databases PubMed, The Cochrane Library and Web of Science from May 2021 to January 2022. A search syntax was developed based on relevant search terms. Various search techniques as well as different operators and combinations (AND, OR, *) were used when entering the search terms (Additional file 1: Table S1). Additionally, the reference lists of the identified studies have been searched for further relevant references.

### Eligibility of studies

In a first step, publications were screened for title and abstract according to inclusion and exclusion criteria individually by the first (PT) and second author (JH). The inclusion criteria comprised (1) DSSs for prescribing antibiotic therapy, (2) DSSs used in hospitals for clinical practice, (3) studies in English with a qualitative, quantitative or mixed-methods study design, (4) published between 2011 and 2021 and (5) examining DSS implementation factors. Exclusion criteria were (1) studies analyzing the technological development of DSSs without practical relevance and (2) DSSs in outpatient care. Studies that seemed to meet the inclusion criteria were selected for a full text review. Second, the full papers of the resulting set of publications was retrieved and reviewed, again by the first (PT) and second author (JH). Any disagreement about the inclusion of a publication between the two reviewers was resolved thorough discussion and, if required, the third author (CD) was consulted.

### Data extraction

The articles were extracted using standardized table formats with the following parameters:

- Authors and publication year
- Country
- Funding of the study
- Study design
- Sample size
- Targeted group
- Characteristics of the DSS
- Methodological quality

**Fig. 1:**
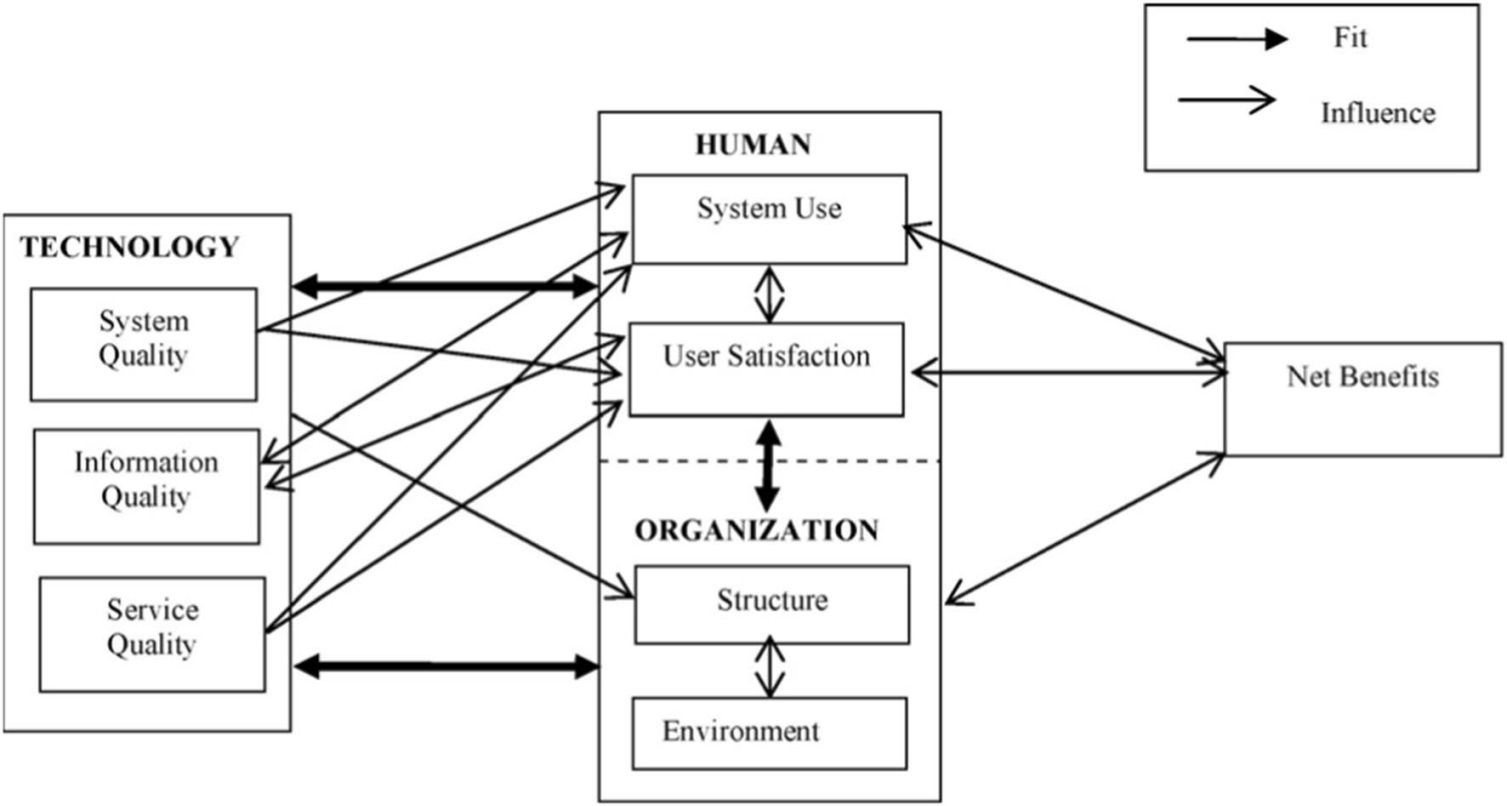
Human–Organization–Technology-fit framework [23]

Despite diverse approaches explaining the implementation of DSSs, the interface between technology-organization-user has been insufficiently considered. To describe and categorize the identified implementation factors, the Human-Organization-Technology-fit-model (HOT-fit model) of Yusof et al. [23] was chosen as a theoretical framework in this paper (Fig. 1).

It focuses the fit between the three domains *technology, human* and *organization* and maps their relationships and possible interactions, as well. The three domains comprise subdomains, so that the factors can be mapped onto seven interrelated dimensions: *System use* and *user satisfaction* in the human domain, *structure* and *environment* in the organizational domain and *system quality*, *information quality* and *service quality* in the technological domain. The framework is completed with the dimension *net benefits*, which comprises the potential negative and positive impacts on individual, organizational or societal level. All these dimensions influence each other in a temporal and causal way. This model has been developed and validated for the implementation and evaluation of innovations in organizations, especially in hospitals [24, 25]. On this basis, not only the static user and system attributes but also dynamic organizational processes that can influence the implementation process will be elaborated. The implementation factors were identified thematically by the first and second author by textual analysis of the included publications. The factors were individually mapped to the HOT-fit dimensions, as described in Table 1. Furthermore, each factor was categorized as either impeding (for example reported on in a publication as hindering or obstructing implementation of DSSs) or facilitating (for example reported on in a publication as positively influencing DSSs implementation). A narrative synthesis was further performed to summarize the evidence on factors most often and less reported respectively.

**Table 1:** Description of the HOT-fit domains [23]

Category		Description
Technology	System quality	System quality measures the inherent features of a system including system performance and user interface. Examples of system quality measures are ease of use, ease of learning, availability, system flexibility, and security
	Information quality	Measures of information quality are concerned with information produced by the system. Criteria that can be used for Information quality are information completeness, accuracy, legibility, timeliness, availability, relevancy, consistency and reliability
	Service quality	Service quality is concerned with the overall support delivered by the service provider of the system and can be measured through quick responsiveness, assurance, empathy and follow up service
Organization	Structure	Organization structure consists of nature including culture, politic, hierarchy, autonomy, planning and support systems, strategy, management and communication. Leadership and top management support can also be measured from the organization factors
	Environment	The environment can be analyzed through its financing source, government, politics, localization, competition, inter-organizational relationship as well as legal regulations
Human	System use	System use relates to the person who uses it, their levels of use, training, knowledge, belief, expectation and acceptance or resistance
	Satisfaction	User satisfaction is often used to measure system success. It is subjective in nature as it depends on whose satisfaction is measured. User satisfaction is defined as the overall evaluation of a user’s experience in using the system and the potential impact of the system. User Satisfaction can be related to user’s perceived usefulness and attitudes towards a system
Net benefits		Net benefits capture the balance of positive and negative impacts on user, which includes clinicians, managers and IT-staff, system developers, hospitals or the entire healthcare sector

### Quality assessment

A critical evaluation of the methodological study quality was performed using the Mixed Methods Appraisal Tool (MMAT) version 2011 [26]. This scoring system is specially developed to concomitantly appraise the methodological quality of qualitative, quantitative and mixed methods studies. The scoring system contains specific quality criteria, which are assessed, if applicable or not. An overall quality score is than calculated as percentage. Publications were scored after the inclusion process by first and second author individually. Disagreement were resolved by consensus. For a better overview and in line with the calculation of the overall score, the quality of the included studies was classified into categories based on the percentages achieved (Table 2).

**Table 2:** Categorization of study quality

Category	Percentage
Good	> 50
Moderate	50
Poor	< 50

## Results

### Study selection

**Fig. 2:**
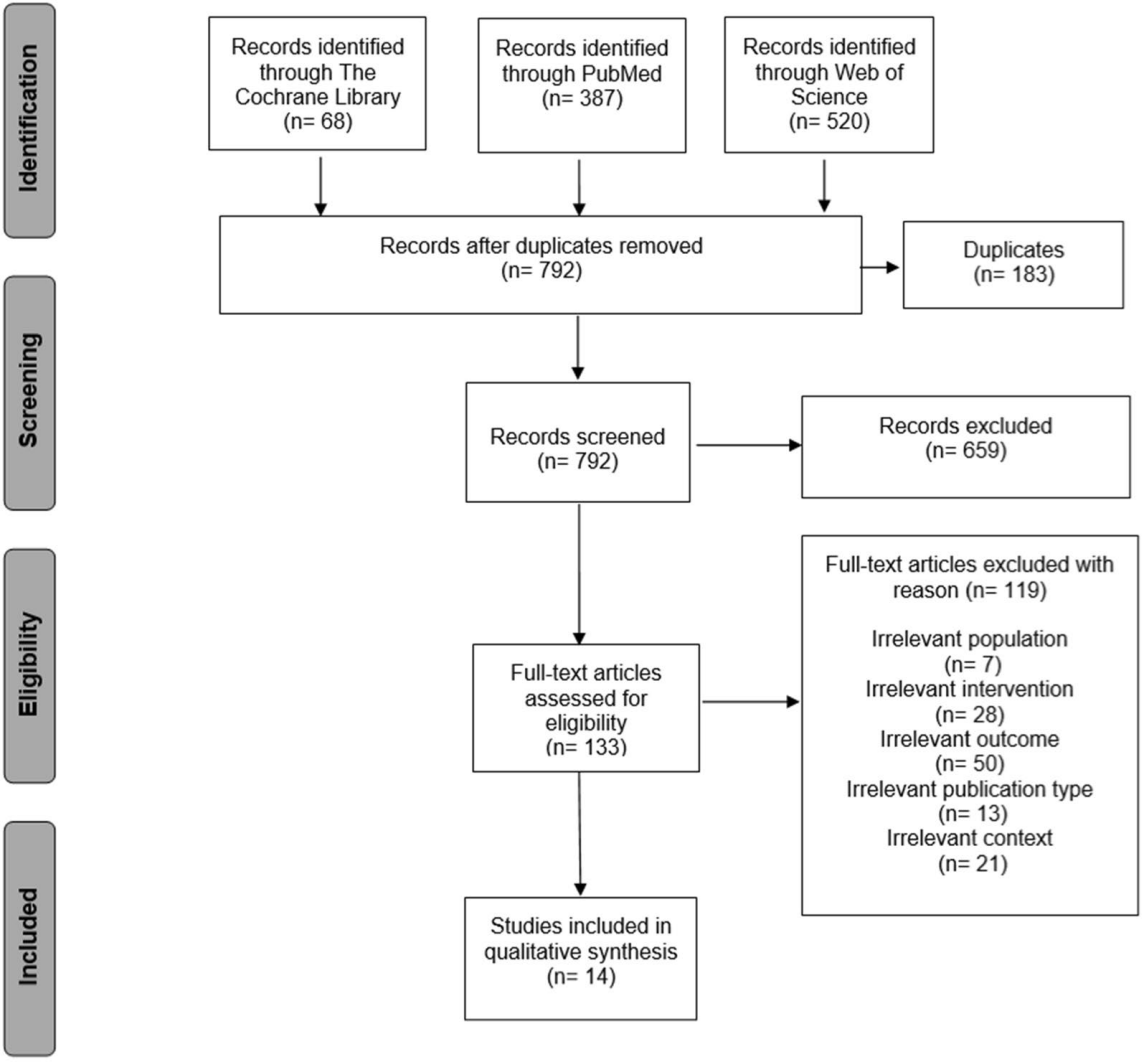
PRISMA flow diagram of literature search and selection process [22]

In total, 975 publications were identified through the databases The Cochrane Library, PubMed and Web of Science (Fig. 2). Duplicates were sorted out and appropriate studies were included in a second screening. 14 publications met the inclusion criteria and were included in the qualitative analysis.

### Study characteristics

A total of five publications were identified from Australia [27–31], two studies from the Netherlands [32, 33] and two publications from Singapore [34, 35]. In addition, one study was conducted in Malaysia [36], one in Sweden [37], one in the United States [38] and in Portugal [39]. One study was conducted concomitantly in France and Switzerland [40]. Most studies (n = 6) used a combination of qualitative and quantitative methods [29, 30, 32, 34, 35, 37] or only qualitative methods (n = 5) [28, 33, 38–40]. Three studies used quantitative methods [27, 31, 36].

In the majority of publications (n = 11) the DSS was fully embedded in existing systems, such as the electronic health record [27–31, 33–36, 39, 40]. In two publications, the DSS was designed as a standalone system that operated independently from existing technical structure [32, 37]. In one study, the DSS characteristics are not described [38]. The quality assessment of the included studies resulted in an average quality score of 75% (range 50–100%). Thereby, all studies fulfilled at least 50% of the MMAT criteria and had at least a moderate quality level. An overview of the quality assessment of included publications (Additional file 2: Table S2) as well as the study characteristics can be found in Additional file 3: Table S3.

### Barriers and facilitators of DSS implementation

A total of 61 factors were identified (Tables 3, 4). Of these, 25 factors could be assigned to the technology-related domain, 15 factors to the organizational domain and eleven factors to the user-related domain. Ten factors included the net benefits of DSSs.

**Table 3:** Facilitating factors for implementation of DSS due to HOT-fit domains

Domain	Theme	Facilitating factors	Study
Technology: system quality	Access/interoperability	Compatibility with existing systems	[27, 32, 34, 35, 37, 39]
		Easy access	[37, 39]
	Layout	Easy navigation (e.g. relevant information texts are easy to find)	[33]
		Relevant functions are visibly placed	[32, 33]
		DSS provides an overview of data and recommendations	[37, 39]
		Results are color highlighted, use of tables and graphs	[37, 39]
	Usability/functions	Important functions (e.g. calculation of the body mass index) are integrated	[28, 32, 33]
		Quick and easy data entry methods	[40]
		Alert functions regarding contraindications, allergy	[32, 39]
	Flexibility/interactivity	DSS are adjustable to specific conditions and patient cases	[32]
		Manual data entry methods for correction and complementation of information (e.g. free text field)	[33, 36, 37]
Technology: information quality	Evidence	Transparent presentation of evidence and comprehensibility of recommendations	[28, 32, 40]
	Currency	Recommendations based on current evidence	[40]
	Clarity	Clear and precise wording of recommendations	[31, 32]
Organization: structure	Participation	Integration of relevant and potential end user groups in planning, development and implementation phase	[27–29, 31, 32, 36, 38, 39]
	Technical support	Support from technical experts	[27, 40]
	Technical equipment	Sufficient number of computers and workstations	[27, 38]
	Multipliers	Integration and support of multipliers and trained persons in implementation phase	[27, 38]
	Training courses	Training courses regarding handling and functions of DSS	[27, 31, 36, 38]
	Internal communikation/feedback	Regular feedback and communication regarding the advantages of DSS use (e.g. antibiotic use, cost savings)	[31]
Organization: environment	Financing	Long-term cost savings due to DSS use	[40]
User: system use	Attitude/opinion	Positive attitude towards technology	[28, 34, 37, 38]
		Positive attitude towards guideline recommendations	[25]
	Experience/familiarity	Prior experiences with DSS	[32, 38]
	Knowledge/competencies	Sufficient technical competences	[36]
Net benefits	Time	Time saving due to structured presentation of data	[28, 32, 40]
	Workflow	Facilitating of workflow and interdisciplinary communication	[32, 35, 37, 39]
		Improvement in treatment quality	[38]
		Guidance in uncertain situations (e.g. in night shifts or for residents)	[28, 34, 35]
	Job autonomy/professional role	Less dependency (e.g. from pharmacists)	[31]
		DSS promote learning process (e.g. regarding scientific research of guideline recommendations)	[28]

**Table 4:** Impeding factors for implementation of DSS due to HOT-fit domains

Domaine	Theme	Impeding factors	Study
Technology: system quality	Access/interoperability	Difficult access to DSS	[29, 39]
		Not compatible with existing systems → double documentation	[38, 39]
	Usability/functions	Complicated data entry methods	[30]
		Alerts are not visible	[29]
		No notification when data has been updated (e.g. new lab results)	[39]
		Data entry insufficiently checked regarding completeness and correctness	[30]
	Flexibility/interactivity	DSS not adjustable to individual conditions and complex patient cases	[27, 28, 30]
		DSS does not provide a holistic approach of the patient cases	[34]
Technology: information quality	Evidence	Recommendations are strictly drawn	[32]
	Completeness	Incomplete information (e.g. regarding local resistance patterns)	[39]
	Relevancy	Insecurity because of irrelevant and too much information and options	[30, 33
Organization: structure	Technical support	Insufficient support regarding technical concerns and questions	27]
	Technical equipment	Insufficient number of computers and workstations	[27, 28]
	Multipliers	Lacking support from management level or multipliers	[28]
	Training courses	Lack of training courses	[27]
	Internal communication/feedback	Lacking knowledge of availability and advantages of DSS	[29]
	Hierarchy/standards	Standards of the unit or team, which are seen as unchangeable and not compatible with new innovations	[28, 34, 35]
		Influence of senior physicians attitude/senior physicians as decision-making authorities	[28, 30, 38]
Organization: environment	Statutory framework	Questions regarding responsibility in medication errors due to DSS use is not regulated by law clearly	[40]
User: satisfaction	Perceived gain	Lack of satisfaction, since there is no perceived gain or benefit	[27, 30]
User: system use	Attitude/opinion	Negative attitude towards technology	[27, 36]
		Resistancy/reservation towards changes	[40]
	Experience/familiarity	Lack of experience with DSS	[27, 28, 33, 40]
	Knowledge/competencies	Idea of not having enough technological competencies for using DSS	[40]
		Lack of knowledge regarding the functions and advantages of DSS	[27, 29]
		Advantages of DSS are seen sceptical of users with more professional experience	[27, 28, 34]
Net benefits	Time	Use of DSS is seen as more time consuming	[27, 38, 40]
	Workflow	Use of DSS means the changeover of work processes	[27, 28, 30, 32, 37, 39, 40]
	Job autonomy/professional role	Physicians tend to rely on DSS only	[40]
		Use of DSS is perceived as an intervention in professional autonomy	[27, 32, 36, 40]

Within the technology domain, with 76% the majority of factors refers to system quality (n = 19/25) and with approx. 24% to information quality (n = 6/25). No factor could be assigned to the area of service quality. The compatibility of the DSS with already existing systems [27, 32, 34, 35, 37, 39] and the flexibility of the system [32, 33, 36, 37] are recognized as facilitating for successful implementation. In contrast, the incompatibility of the DSS with existing systems and the resulting double documentation [38, 39] as well as a complicated access to the DSS [29, 39] are described as impeding.

About 87% of the organizational factors (n = 13/15) can be assigned to structural conditions and about 13% to the organizational environment (n = 2/15). With 57% the majority of publications (n = 8/14) examines factors related to participation and the integration of potential user groups in the planning, development and implementation phases as facilitating for implementation [27–29, 31, 32, 36, 38, 39]. Moreover, the attitude of senior physicians in particular is crucial, so that a negative attitude can have a negatively impact on the implementation of DSSs in practice [28, 30, 38]. On the other hand, insufficient technical equipment or workstations represent impeding aspects [27, 28].

In the human domain, about 91% (n = 10/11) of the factors relate to system use and only two factors to user satisfaction (about 9%). In this context, a positive attitude towards technologies is positively associated with the use of the technology [28, 34, 37, 38] while a negative attitude imped successful implementation [27, 36]. Moreover, prior experience with DSSs is positively associated with successful implementation [32, 38], while insufficient familiarity with DSSs leads to less acceptance and not successful implementation [27, 28, 33, 40]. Additionally, a lack of satisfaction impedes the successful implementation of DSSs [27, 30].

Apart from these three domains, twelve publications examine positive and negative effects of DSS implementation [27, 28, 30–32, 34–40]. The factors predominantly relate to the impact of DSSs on workflow [27, 28, 30, 32, 34, 35, 37–40], perceptions of professional autonomy [27, 28, 31, 32, 36, 40] and time constraints [27, 28, 32, 38, 40].

## Discussion

### Main findings

In undertaking this review, we have provided an overall picture of the current evidence surrounding existing factors to promote the implementation of DSSs for antibiotic prescription in hospitals. Multiple factors could be identified that might affect the success or failure of DSS implementation. According to the HOT-fit framework, the majority of factors could be assigned to the domain of technology and organization.

Clearly, a successful DSS is dependent on the completeness and accuracy of the evidence base used to support it and the technical design of the system modalities. However, DSS is not just about technical content or technical design; DSSs involve workflow. DSSs are as much an organizational as a technical intervention, and organizational, professional and other challenges to implementing DSSs may be as daunting as the technical challenges [41]. The findings of this review show that intraorganizational standards or rules might not be compatible with the implementation of new technologies like DSSs and that members of the organization first have to be convinced from potential advantages of DSSs. Adopting a new technology is often about getting out of comfort zone and laying hands on to new things that require some extra effort, which can be challenging both for organizations as well as the individuals being a part of the organization [41].

Thus, factors concerning the potential impact of implementing DSSs, such as the time required or integration into existing workflow are assessed as fundamental to the implementation process. A disrupted workflow can lead to increased cognitive effort, more time required to prescribe and less time face-to-face with patients [42]. How to integrate DSSs with clinicians´ workflow, however, remains a challenge, in part because there are no current standards for clinical workflow. With regard to organizational factors, this review points out that it is crucial to pay attention to the social context i.e. the hospital setting when designing and implementing DSSs. This requires the need for developing strategies that consider the organizational structures and the specific roles of potential target groups in this social context. In addition, the success of DSSs is determined by the policies, norms and culture of the organization in which they are being used [43]. In fact, special attention should be paid to the integration and implementation of DSSs in hospitals, as well as their adoption and utilization by clinicians. Co-design including clinicians and system developers may be key for success and allow to study the interaction between health professionals and DSSs and promote the implementation in clinical practice [44]. To enable DSSs to improve clinical workflow, the use of user-centered design principles and techniques during the initial design phase seem crucial. In particular, determining needs rather than user desires is an important consideration.

This work can be taken forward as a basis for designing and integrating DSSs for antibiotic prescription in a hospital setting, since central components of such interventions need to build on the existing literature, as identified in this work and on existing guidance surrounding the development and evaluation of complex interventions [45]. Ideally, future work should consist of multicenter randomized controlled trials. Embedding qualitative evaluation would ensure that end-user perspectives are considered properly. Given the complex effects of DSSs and variety of settings in which they are used, randomized controlled trials may not be feasible, in which case quasi-experimental studies may be considered. In this context, paying attention to both social and technical dimensions of change as well as drawing on longitudinal qualitative designs for integration user perspective is central for going forward [46].

In relation to systems design there are various options that can help to promote the appropriate use of antibiotics. These include the availability of large volumes of electronic data, which allows the provision of reliable recommendations and so increases the trustworthiness of the DSS. Moreover, an easy access to the DSS and understandable provision of relevant data are crucial. As described in the most of the publications [27, 32, 34, 35, 37, 39] embedding DSS in existing technical structures, like electronic health records or hospital information systems can yield synergistic effects in improving implementation process, as additional effort to learn to operate with the new technical infrastructure is minimized [42, 47, 48].

In any case, appropriate organizational and social components will need to play an essential part. For this reason, it is important to identify stakeholders that support implementation and a multidisciplinary team to achieve realization, distribution and continuation. The literature synthesis also showed that training with the new system and the availability of educational material are important contributors to successful implementation [49]. In light of these findings, clinicians should receive enhanced and hands-on training prior to implementation to familiarize them as much as possible with the system before it is actually implemented in daily practice [48].

Sociotechnically guided work can also help to ensure the improvement in clinicians’ performance regarding antibiotic prescription effectively. Here, examination the interaction of technical features and user interfaces as well as organizational aspects such as training will be crucial. User perspectives and needs are equally important factors in DSS implementation, which are closely related to the requirement to involve potential user groups early in the development process. For the successful development and implementation of new technical systems it is also essential that various stakeholders are able to integrate their different knowledge and perspectives in this process. By involving relevant target groups, a positive attitude towards advantages and the benefits of DSSs can arise [47].

As highlighted in this review, DSS facilitates decision-making and thus reduces the burden on clinicians. However, when used regularly, the degree of reliance or trust on DSS can also generate a form of dependency. Additionally, the user’s ability to act may be limited, when the system fails [41]. Also, the risk of “deskilling” clinicians who use DSSs and the importance of minimizing the perceived threats to professional autonomy are commonly cited in previous reviews [42, 50–52], as well as in this review. In this context, trust is an important success factor, so that traceability and transparency should be created when using DSSs. Trust is a social construction that originates from interpersonal relationships [53]. Due to its relationship-based nature, it has been argued that trust is multifaceted and that the formation of trusting beliefs involves conjunctive underlying reasons [54]. Prior research suggests that trust can be attributed to a variety of causes, such as dispositional, calculative, institutional and knowledge-based reasons [53]. A DSS, that informs users about how and why it performs in a certain way, enables users to better understand its recommendations [55]. It should provide the necessary knowledge and guidance for them to make informed choices when prescribing antibiotics. Additionally, institution-based trust is crucial for trust formation. It is associated with societal structures, that represent institutional safeguards, on which the members of the institution can rely on. Structural assurance is an assessment of trust based on contextual conditions, e.g., regulations, involvement and legal guidelines [53]. Moreover, trust in technologies influences persons´ use of technology and their perception of the advantages provided by these technologies [55]. Users´ expectation about DSS design and performance can influence trust in DSS due to an interactive reason. Understanding trust formation in DSS will assist researchers and practitioners in identifying the design that augments users´ trust and supporting their professional abilities concurrently, which will consequently increase the acceptance of DSSs. Future research is therefore recommended on DSS design that can enhance users´ trust in DSSs for antibiotic prescription.

The likely difficulty for any future work in this area is the complexity of DSSs. These are likely to consist of multiple components accompanied by organizational characteristics. This justifies the high importance of future research integrating the user perspective. Embedded qualitative designs can help to facilitate insights into which components are potentially transferable between settings for antibiotic prescribing. Such work is also needed to gain deeper insights into the impact on time and reservations amongst users as well as other reasons as to why DSS implementation for antibiotic prescription is not successful [56].

### Strength and limitations

The mixed method synthesis approach, which has been used to identify factors for DSS implementation has several strength. First, due to the large variety in the methods used in research, combining the results of quantitative, qualitative and mixed methods studies was well suited for this review. The multilevel approach chosen for this review was expedient, since different types of factors, which are important for DSS implementation for antibiotic prescription in hospitals, were considered. We, however, considered publications published in the last decade, so that only studies on current generation of DSSs were included. Third, we applied a systematic approach for data extraction with two independent researcher assessing the inclusion criteria per publication, extracting implementation factors and mapping these to the dimensions of the HOT-fit framework. However, several of the identified factors- impeding as well as facilitating- are not unique to DSSs for antibiotic prescribing. Nevertheless, findings from this review also highlight factors that are specific to antibiotic prescription in hospitals and should be considered. For instance, guidance and recommendations for antibiotic treatment require in most cases more detailed information about patient history, previous antibiotic exposure, local resistance patterns and proper examination of microbiological test results. The provision of flexible and comprehensible recommendations appears to be an important factor to increase the uptake of DSSs like highlighted in this review. Indeed, several studies have reported a correlation between DSS adoption and their impact on antibiotic prescription [57, 58], which highlights the need to assess not only the effects of DSSs on antibiotic prescription but also their implementation process. In order to optimize DSS implementation for antibiotic prescription, current gaps in implementation and evaluation should be addressed, which comprises, inter alia, user satisfaction and the impact on clinicians´ attitude in line with specific organizational characteristics. In fact, this review might be considered as a basis for further research of DSS implementation in the field of antibiotic prescribing in hospitals.

The interpretation of the results of this review and their representativeness requires the consideration of some methodological limitations. Despite the intensive efforts, it is possible that not all relevant studies have been identified as some may not be available in the public domain, and others may be published outside the peer-reviewed academic literature. Furthermore, the MMAT has been used to assess the methodological quality of the studies. One advantage of this assessment tool is the easy applicability, since criteria are assessed, which are only dichotomously differentiated. However, this leads to the major disadvantage that only a rough differentiation of the quality assessment of the included studies can be made. Final limitation of this review is that the HOT-fit framework was considered useful; the mapping of factors to the HOT-fit framework was a subjective undertaking of two independent researchers. Some factors proved to be difficult to map to one specific evaluation measure of a certain dimension, because these evaluation measures were sometimes not clearly defined or ambiguous. Additionally, the mapping does not reflect the level of interaction between the various factors and their relative importance. Importantly, the findings may not necessarily indicate the significance of a particular issue. Rather, the relative weight of these factors should be determined in developing and implementing DSSs. Nevertheless, the consideration of the HOT-fit framework was expedient to systemize implementation factors and to highlight the importance of paying attention to technical components and the users as well as the surrounding environment for the implementation process all together [59]. Furthermore, by using sociotechnical frameworks during the development and implementation phase of DSSs, possible impeding or facilitating factors can be identified in an early stage and so appropriate strategies can be defined to increase the success of DSS implementation for antibiotic prescription in hospitals.

## Conclusion

There is yet inconclusive evidence about the factors influencing the implementation of DSSs for antibiotics prescription in hospitals. The wide range of identified impeding and facilitating factors contribute to the understanding of the implementation process and suggest that there is no “one size fits all approach”. In future, there is a clear need to pay closer attention to social as well as human components and with a broader organizational focus when developing and implementing DSSs for antibiotic prescription in hospitals.

## Supplementary Information

**Additional file 1. Table S1.** Search strategy.

**Additional file 2. Table S2.** Appraisal of the methodological quality.

**Additional file 3. Table S3.** Study characteristics.

## Data Availability

All data generated or analyzed during this study are included in this published article and its Additional files 1, 2 and 3.

## Abbreviations

DSS: Decision support system
HOT-fit-model: Human–Organization–Technology-fit-model
MMAT: Mixed Methods Appraisal Tool
