# Supplementary material for: Factors influencing the implementation of decision support systems for antibiotic prescription in hospitals: a systematic review
Source: BMC Med Inform Decis Mak. 2023 Feb 6;23:27. doi: 10.1186/s12911-023-02124-4 (PMC9903563; doi:10.1186/s12911-023-02124-4)
Supplement: Supplementary file 1 — Additional file 1. Table S1. Search strategy. [file 12911_2023_2124_MOESM1_ESM.docx]

Table 1: Search strategy

| Search terms/combination |
| --- |
| #1 empiric antibiotic therapy  #2 antibiot*  #3 antimicrob*  #4 #1 OR #2 OR #3  #5 decision support system  #6 clinical decision support  #7 electronic prescribing  #8 DSS  #9 #5 OR #6 OR #7 OR #8  #10 hospital  #11 inpatient*  #12 clinical setting  #13 secondary care  #14 #10 OR #11 OR #12 OR #13  #15 #4 AND #9 AND #14  #16 implement*  #17 implementation factors  #18 #16 OR #17  #19 #15 AND #18  #20 #19 AND Filters: 2011 to 2021 |
