# Supplementary material for: Factors influencing the implementation of decision support systems for antibiotic prescription in hospitals: a systematic review
Source: BMC Med Inform Decis Mak. 2023 Feb 6;23:27. doi: 10.1186/s12911-023-02124-4 (PMC9903563; doi:10.1186/s12911-023-02124-4)
Supplement: Supplementary file 2 — Additional file 2. Table S2. Appraisal of the methodological quality. [file 12911_2023_2124_MOESM2_ESM.docx]

Table 2: Appraisal of the methodological quality

| **Methodological quality criteria** | Zaidi et al. (2012)  [27] | Zaidi et al. (2013)  [28] | Diasonis et al.  (2015)  [29] | Baysari et al.  (2017)  [30] | | Chavada et al.  (2017)  [21] | | Beerlage- de Jong et al. (2016)  [32] | | Akhloufi et al. (2019)  [33] |
| --- | --- | --- | --- | --- | --- | --- | --- | --- | --- | --- |
| Are there clear qualitative and quantitative research questions (or objectives*), or a clear mixed methods question (or objective*)? | ✓ | ✓ | ✓ | ✓ | | ✓ | | ✓ | | ✓ |
| Do the collected data allow address the research question (objective)? | ✓ | ✓ | ✓ | ✓ | | ✓ | | ✓ | | ✓ |
| **Qualitative** | | | | | | | | | | |
| Are the sources of qualitative data (archives, documents, informants, observations) relevant to address the research question (objective)? |  | ✓ | ✓ | ✓ | | X | | ✓ | | ✓ |
| Is the process for analyzing qualitative data relevant to address the research question (objective)? |  | ✓ | ✓ | ✓ | | ✓ | | ✓ | | ✓ |
| Is appropriate consideration given to how findings relate to the context, e.g., the setting, in which the data were collected? |  | ✓ | ✓ | ✓ | | ✓ | | ✓ | | ✓ |
| Is appropriate consideration given to how findings relate to researchers’ influence, e.g., through their interactions with participants? |  | ✓ | X | ✓ | | X | | X | | X |
| **Quantitative, randomized controlled trials** | | | | | | | | | | |
| Is there a clear description of the randomization (or an appropriate sequence generation)? |  |  |  |  | |  | |  | |  |
| Is there a clear description of the allocation concealment (or blinding when applicable)? |  |  |  |  | |  | |  | |  |
| Are there complete outcome data (80% or above)? |  |  |  |  | |  | |  | |  |
| Is there low withdrawal/drop-out (below 20%)? |  |  |  |  | |  | |  | |  |
| **Quantitative, nonrandomized** | | | | | | | | | | |
| Are participants (organizations) recruited in a way that minimizes selection bias? |  |  |  | ✓ | |  | |  | |  |
| Are measurements appropriate (clear origin, or validity known, or standard instrument; and absence of contamination between groups when appropriate) regarding the exposure/intervention and outcomes? |  |  |  | X | |  | |  | |  |
| In the groups being compared (exposed vs. non-exposed; with intervention vs. without; cases vs. controls), are the participants comparable, or do researchers take into account (control for) the difference between these groups? |  |  |  | ✓ | |  | |  | |  |
| Are there complete outcome data (80% or above), and, when applicable, an acceptable response rate (60% or above), or an acceptable follow-up rate for cohort studies (depending on the duration of follow-up)? |  |  |  | ✓ | |  | |  | |  |
| **Quantitative, descriptive** | | | | | | | | | | |
| Is the sampling strategy relevant to address the quantitative research question (quantitative aspect of the mixed methods question)? | ✓ |  | ✓ |  | |  | | ✓ | |  |
| Is the sample representative of the population understudy? | ✓ |  | ✓ |  | |  | | ✓ | |  |
| Are measurements appropriate (clear origin, or validity known, or standard instrument)? | ✓ |  | X |  | |  | | X | |  |
| Is there an acceptable response rate (60% or above)? | X |  | X |  | |  | | ✓ | |  |
| **Mixed methods** | | | | | | | | | | |
| Is the mixed methods research design relevant to address the qualitative and quantitative research questions (or objectives), or the qualitative and quantitative aspects of the mixed methods question (or objective)? |  |  | ✓ | ✓ | |  | | ✓ | |  |
| Is the integration of qualitative and quantitative data (or results*) relevant to address the research question (objective)? |  |  | ✓ | ✓ | |  | | ✓ | |  |
| Is appropriate consideration given to the limitations associated with this integration, e.g., the divergence of qualitative and quantitative data (or results*) in a triangulation design? |  |  | X | ✓ | |  | | X | |  |
| **Overall quality score** | | | | | | | | | | |
|  | 75% (***) | 100% (****) | 50% (**) | 75% (***) | | 50% (**) | | 75% (***) | | 75% (***) |
| **Methodological quality criteria** | Chow et al. (2015)  [34] | Chow et al. (2016)  [35] | Esmaeil-zadeh et al.  (2015)  [36] | Forsmann et al.  (2013)  [37] | | Giuliano et al.  (2018)  [38] | | Simões et al. (2018)  [39] | | Catho et al.  (2020)  [40] |
| **Screening questions (for all types)** | | | | | | | | | | |
| Are there clear qualitative and quantitative research questions (or objectives*), or a clear mixed methods question (or objective*)? | ✓ | ✓ | ✓ | | ✓ | | ✓ | ✓ | ✓ | |
| Do the collected data allow address the research question (objective)? | ✓ | ✓ | ✓ | | ✓ | | ✓ | ✓ |  | |
| **Qualitative** | | | | | | | |  |  | |
| Are the sources of qualitative data (archives, documents, informants, observations) relevant to address the research question (objective)? | ✓ | ✓ | ✓ | | ✓ | | ✓ | ✓ | ✓ | |
| Is the process for analyzing qualitative data relevant to address the research question (objective)? | ✓ | X | X | | ✓ | | ✓ | X | ✓ | |
| Is appropriate consideration given to how findings relate to the context, e.g., the setting, in which the data were collected? | ✓ | ✓ | ✓ | | ✓ | | ✓ | ✓ | ✓ | |
| Is appropriate consideration given to how findings relate to researchers’ influence, e.g., through their interactions with participants? | ✓ | X | X | | ✓ | | ✓ | X | ✓ | |
| **Quantitative, randomized controlled trials** | | | | | | | |  |  | |
| Is there a clear description of the randomization (or an appropriate sequence generation)? |  |  |  | |  | |  |  |  | |
| Is there a clear description of the allocation concealment (or blinding when applicable)? |  |  |  | |  | |  |  |  | |
| Are there complete outcome data (80% or above)? |  |  |  | |  | |  |  |  | |
| Is there low withdrawal/drop-out (below 20%)? |  |  |  | |  | |  |  |  | |
| **Quantitative, nonrandomized** | | | | | | | |  |  | |
| Are participants (organizations) recruited in a way that minimizes selection bias? |  |  |  | |  | |  |  |  | |
| Are measurements appropriate (clear origin, or validity known, or standard instrument; and absence of contamination between groups when appropriate) regarding the exposure/intervention and outcomes? |  |  |  | |  | |  |  |  | |
| In the groups being compared (exposed vs. non-exposed; with intervention vs. without; cases vs. controls), are the participants comparable, or do researchers take into account (control for) the difference between these groups? |  |  |  | |  | |  |  |  | |
| Are there complete outcome data (80% or above), and, when applicable, an acceptable response rate (60% or above), or an acceptable follow-up rate for cohort studies (depending on the duration of follow-up)? |  |  |  | |  | |  |  |  | |
| **Quantitative, descriptive** | | | | | | | |  |  | |
| Is the sampling strategy relevant to address the quantitative research question (quantitative aspect of the mixed methods question)? | ✓ | ✓ | ✓ | | ✓ | |  |  |  | |
| Is the sample representative of the population understudy? | ✓ | ✓ | ✓ | | ✓ | |  |  |  | |
| Are measurements appropriate (clear origin, or validity known, or standard instrument)? | ✓ | ✓ | ✓ | | ✓ | |  |  |  | |
| Is there an acceptable response rate (60% or above)? | X | X | ✓ | | X | |  |  |  | |
| **Mixed methods** | | | | | | | |  |  | |
| Is the mixed methods research design relevant to address the qualitative and quantitative research questions (or objectives), or the qualitative and quantitative aspects of the mixed methods question (or objective)? | ✓ | ✓ | ✓ | | ✓ | |  |  |  | |
| Is the integration of qualitative and quantitative data (or results*) relevant to address the research question (objective)? | ✓ | ✓ | ✓ | | ✓ | |  |  |  | |
| Is appropriate consideration given to the limitations associated with this integration, e.g., the divergence of qualitative and quantitative data (or results*) in a triangulation design? | ✓ | X | X | | ✓ | |  |  |  | |
| **Overall quality score** | | | | | | | |  |  | |
|  | 75% (***) | 50% (**) | 100% (****) | | 75% (***) | | 100% (****) | 50% (**) | 100% (****) | |
| **Legend:** ✓: Applicable, X: Not applicable, -: unknown /: 0% of the criteria were applicable, *: 25%: of the criteria were applicable, **: 50% of the criteria were applicable, ***: 75% of the criteria were applicable, ****: 100% of the criteria were applicable | | | | | | | | | | |
