# Supplementary material for: Factors influencing the implementation of decision support systems for antibiotic prescription in hospitals: a systematic review
Source: BMC Med Inform Decis Mak. 2023 Feb 6;23:27. doi: 10.1186/s12911-023-02124-4 (PMC9903563; doi:10.1186/s12911-023-02124-4)
Supplement: Supplementary file 3 — Additional file 3. Table S3. Study characteristics. [file 12911_2023_2124_MOESM3_ESM.docx]

Table 3: Study characteristics

| **Study** | **Country** | **Funding** | **Study design** | **Sample size** | **Target group** | **Characteristics of the decision support system** | **Study quality** |
| --- | --- | --- | --- | --- | --- | --- | --- |
| Zaidi & Marriott (2012)  [27] | Australia | State | Quantitative study | N= 111 | Physicians  (Interns and senior physicians) and pharmacists | - Web application (Guidance DS®) for electronic prescribing of antimicrobials with integrated DSS (iApprove®)  - Recommendations are made in line with national guidelines  🡪 patient information and data has to be entered manually | *** |
| Zaidi & Thursky (2013)  [28] | Australia | State | Qualitative  study | N= 42 | Physicians  (Interns and senior physicians) and pharmacists | - Web application (Guidance DS®) for electronic prescribing of antimicrobials with integrated DSS (iApprove®)  - Recommendations are made in line with national guidelines  🡪 patient information and data has to be entered manually | **** |
| Diasonis et al.  (2015)  [29] | Australia | State | Mixed-methods-study | Nquali= 12  Nquanti= 545 | Physicians  (Interns and senior physicians) | - TDM= Therapeuting Drug Monitoring  🡪 Web application for medication administration and prescribing  🡪 Integrated MedChart: computerized alters when a specific antimicrobial is indicated, as well as dosing recommendation and instructions for use | ** |
| Baysari  et al.  (2017)  [30] | Australia | State | Mixed-methods-study | Nquali= 11  Nquanti= n. i. | Physicians  (Interns and senior physicians) | - CPOE: Software system, that allows electronic prescribing, pharmacy review and medication administration  - CPOE with integrated DSS  🡪 System interfaces with other hospital clinical information systems, including EHR, so that patient information could automatically be extracted and also combined with local resistance patterns  - Recommendations are in line with national guidelines  🡪 Computerized alert when an inappropriate prescription has been made | *** |
| Chavada et al.  (2017)  [31] | Australia | State | Quantitative study | N= 299 | Physicians  (Interns and senior physicians) | - Within the antibiotic stewardship program antimicrobials were categorized into tiers of restriction using a traffic light system  - As a part of this a pre-prescription approval via an DSS, that were integrated in the EHR, was made | ** |
| Beerlage- de Jong et al.  (2016)  [32] | The Netherlands | State | Mixed-methods-study | Nquali= 6  Nquanti= 54 | Physicians  (Interns) | - Physicians were presented with a scenario-based prototype example of what a DSS might look like  - DSS as a web application and stand-alone-system  🡪 relevant patient information were automatically extracted from the EHR, whereby some additional information could to be entered manually.  - Recommendations are in line with national guidelines  - Participants were integrated in the system development phase | *** |
| Akhloufi et al.  (2019)  [33] | The Netherlands | State | Qualitative study | N= 8 | Physicians  (Interns) | - DSS as a web application and stand-alone-system  🡪 Relevant patient information were as much as possible automatically extracted from the electronic health record (EHR), whereby some information had to be entered manually  - The DSS combines relevant information with relevant local antibiotic treatment guidelines, which are in line with national guidelines  🡪 Rule- and indication-based prescription of antibiotic treatment | *** |
| Chow  et al.  (2015)  [34] | Singapore | N.i. | Mixed-methods-study | Nquali= 11  Nquanti= 265 | Physicians  (Interns and senior physicians) | - Medication administration was made via a CPOE-system that interfaces with the HER  - 2009 a DSS was integrated to the CPOE-system  🡪 relevant patient information were automatically extracted from the EHR  - Recommendations are in line with national guidelines | *** |
| Chow  et al.  (2016)  [35] | Singapore | N.i. | Mixed-methods-study | Nquali= k. A.  Nquanti= 56993 patient cases | Physicians  (Interns and senior physicians) | - Medication administration was made via a CPOE-system that interfaces with the EHR  - 2009 a DSS was integrated to the CPOE- system  🡪 relevant patient information were automatically extracted from the EHR  - Recommendations are in line with national guidelines | ** |
| Esmaeil-zadeh  et al.  (2015)  [36] | Malaysia | State | Quantitative study | N= 306 | Physicians  (Interns and senior physicians) | - DSS integrated in the EHR, so that recommendations are made based on relevant patient information and in line with national guidelines | **** |
| Forsmann et al. (2013)  [37] | Sweden | State | Mixed-methods-study | Nquali= 9  Nquanti= 8 | Physicians  (Interns and senior physicians) | - DSS as a web application and stand-alone-system  🡪 Relevant patient information were as much as possible automatically extracted from the electronic health record (EHR), whereby some information could to be entered manually  - the systems gives a brief overview of patient cases by highlighting relevant and important data and information as well as resistance patterns  - Recommendations in line with national guidelines | *** |
| Giuliano et al.  (2018)  [38] | USA | No funding | Qualitative  study | N= 19 | Pharmacists | - A rule-based DSS was developed along with pharmacists and physicians  - No further information regarding functions, layout and so on | **** |
| Simões  et al.  (2018)  [39] | Portugal | State | Qualitative  study | N. i. | Physicians and the Antibiotic Stewardship-Team | - HaiTool  🡪 Monitoring-tool with integrated DSS  🡪 web application for medication administration and prescription with integrated DSS  🡪 System bundles relevant patient information and resistance patterns  🡪 based on this and in line with national guidelines recommendations are made | ** |
| Catho  et al.  (2020)  [40] | Switzerland/  France | State | Qualitative  study | N= 27 | Physicians  (Interns and senior physicians) | - DSS were integrated in the EHR, that also interfaced with a COPE-system  🡪 based on this all relevant information were bundled and an appropriate recommendation could be made | **** |
| Abbreviations and legend: EHR: Electronic Health Record, DSS: Decision Support System, CPOE: Computerized Physician Order Entry, TDM: Therapeuting Drug Monitoring; /: 0% of the quality criteria were applicable, *: 25% of the quality criteria were applicable, **: 50% of the quality criteria were applicable, ***: 75% of the quality criteria were applicable, ****: 100% of the quality criteria were applicable, n. i.: no information. | | | | | | | |
